# Supplementary material for: Organic photodiodes with bias-switchable photomultiplication and photovoltaic modes
Source: Nat Commun. 2023 Oct 31;14:6935. doi: 10.1038/s41467-023-42742-0 (PMC10618528; doi:10.1038/s41467-023-42742-0)
Supplement: Supplementary file 1 — Supplementary Information [file 41467_2023_42742_MOESM1_ESM.pdf]

Supplementary information

Organic Photodiodes with Bias-Switchable Photomultiplication and Photovoltaic Modes

Qingxia Liu[1], Lingfeng Li[1], Jiaao Wu[1], Yang Wang[1]*, Liu Yuan[1], Zhi Jiang[2], Jianhua Xiao[1], Deen Gu[1], Weizhi Li[1], Huiling Tai[1]*, and Yadong Jiang[1]

[1]State Key Laboratory of Electronic Thin Films and Integrated Devices, School of Optoelectronic Science and Engineering, University of Electronic Science and Technology of China, Chengdu 610054, China.
[2]Innovative Center for Flexible Devices (iFLEX), School of Materials Science and Engineering, Nanyang Technological University, 50 Nanyang Avenue, Singapore 639798, Singapore.
*Email: landlord@uestc.edu.cn; taitai1980@uestc.edu.cn

Supplementary Discussion

The specific detection (D^*) of a photodetector is one of the most important figure-of-merits that determines the sensitivity of a photodetector to optical signals and can be calculated by

$$D^* = \frac{R\sqrt{AB}}{I_n} = \frac{R\sqrt{A}}{S_n} \ (Jones) \qquad (1)$$

where R is the responsivity, A is the active device area, B is the bandwidth, I_n is the noise current and S_n is the noise current spectral density. When the device noise is dominated by the shot noise, D^* can be obtained by

$$D^* = \frac{R\sqrt{A}}{\sqrt{2qi_d}} = \frac{R}{\sqrt{2qJ_d}} \ (Jones) \qquad (2)$$

where q, $i_{d,}$ and J_d are the electron charge, dark current, and dark current density, respectively. Thus, low J_d and high R are desirable for superior D^*.

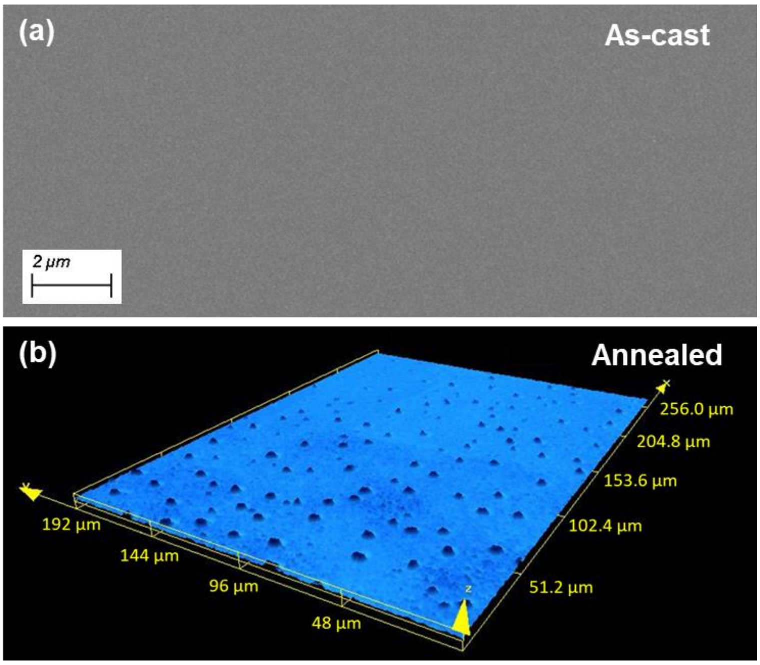

Supplementary Fig. 1 a SEM image of the as-cast BHJ film. **b** 3D morphology of an annealed BHJ film (150-nm-thick, annealed at 250°C for 60 min) measured by laser scanning confocal microscope.

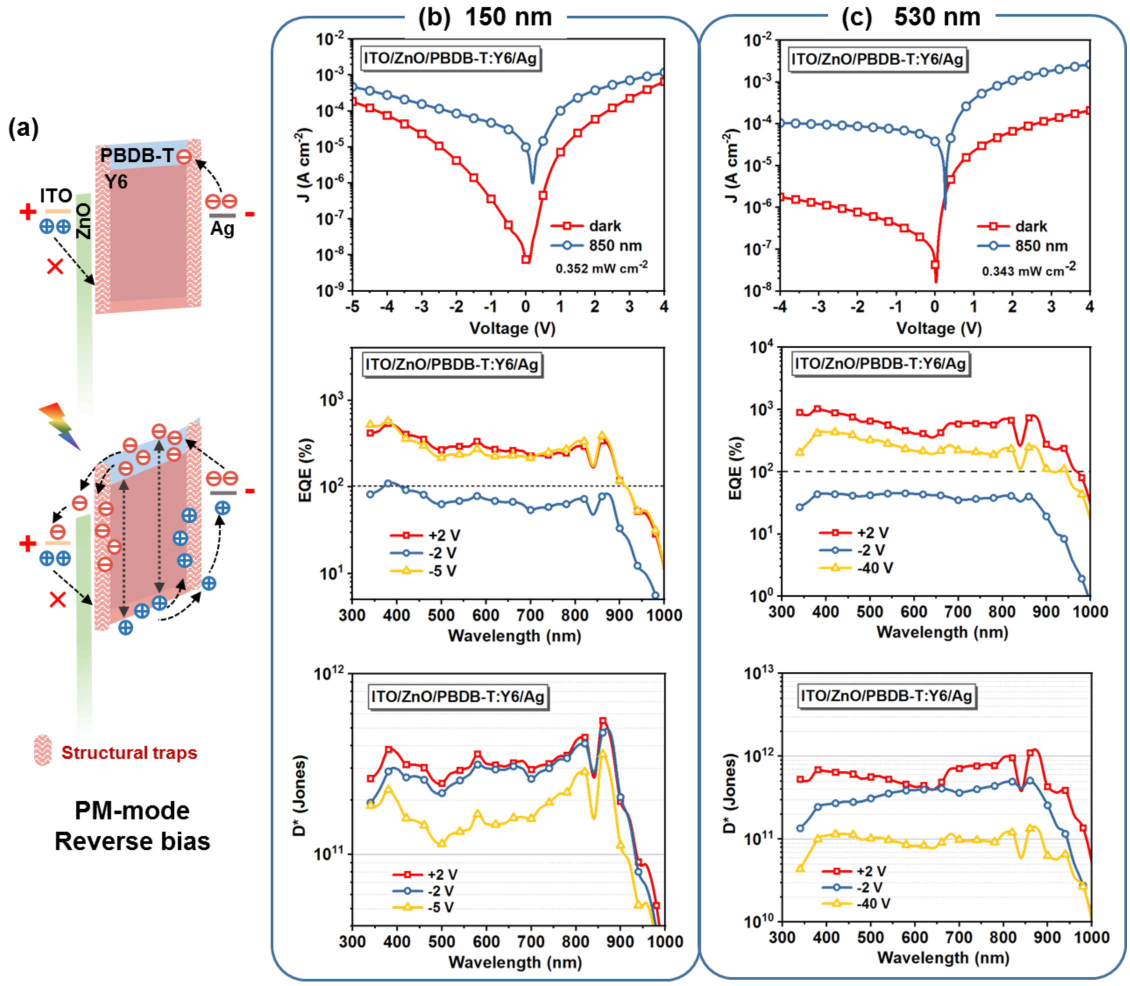

Supplementary Fig. 2 **a** Schematic diagrams illustrating the working mechanisms of the MoO_3-free OPD operated under reverse bias. *J*-*V* curves in dark and under 850 nm monochromatic illumination (0.343 mW cm^{-2}), EQE and *D** spectra of the **b** 150-nm-thick and **c** 530-nm-thick devices operated under different biases.

The structure of the MoO_3-free OPDs are both ITO/ZnO/PBDB-T:Y6 (30-min-annealed at 250°C)/Ag. For the 150-nm-thick device, the PV-mode cannot be maintained at a low bias of only -2 V, as the EQE values exceed 100% in the range of 360-400 nm. When the bias is increased to -5 V, the EQE values in the whole measured range are almost the same as the PM-mode of +2 V. For the 530-nm-thick device, the photocurrent of the device is suppressed under reverse bias due to the thick active layer, thus a high bias is required to induce band bending and charge tunneling injection (-40 V in this case). The results verify the importance of MoO_3 blocking layer for maintaining PV-mode under reverse bias.

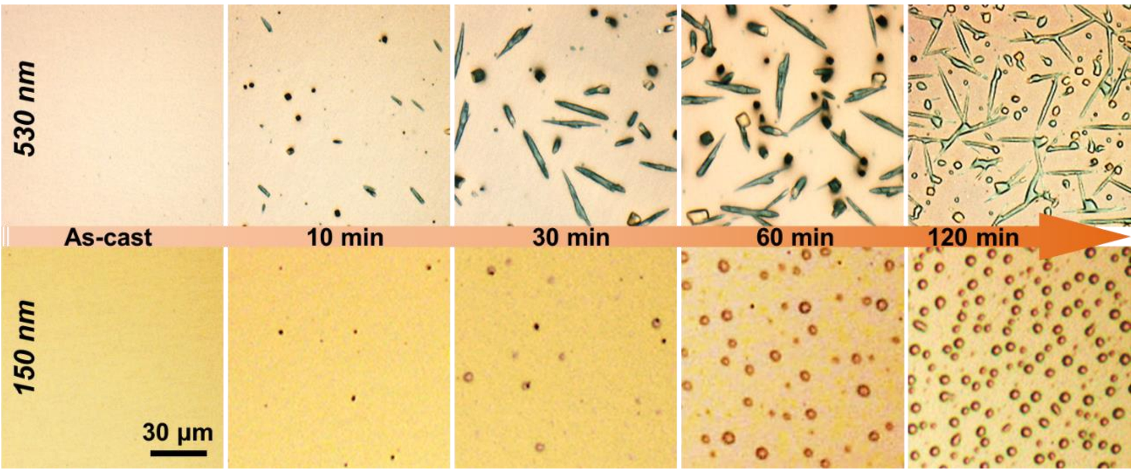

Supplementary Fig. 3 Surface micrographs of PBDB-T:Y6 BHJ films annealed at 250°C for different durations, measured by laser scanning confocal microscope. The thicknesses of BHJ films are 150 nm and 530 nm. The scale bar is equal for all micrographs.

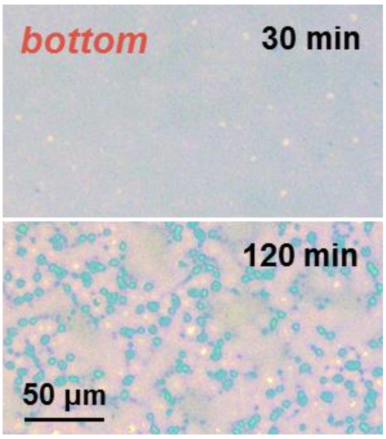

Supplementary Fig. 4 Optical micrographs for the bottom side of 530-nm-thick BHJ films, which were annealed at 250°C for 30 min and 120 min.

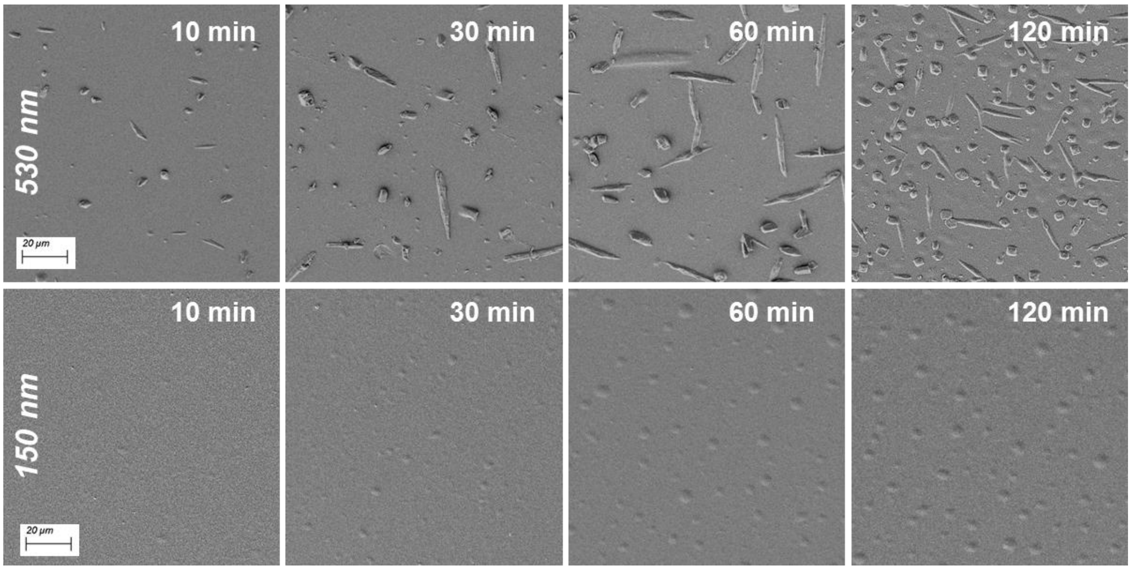

Supplementary Fig. 5 30°-tilted SEM images of the BHJ films annealed at 250°C for different durations.

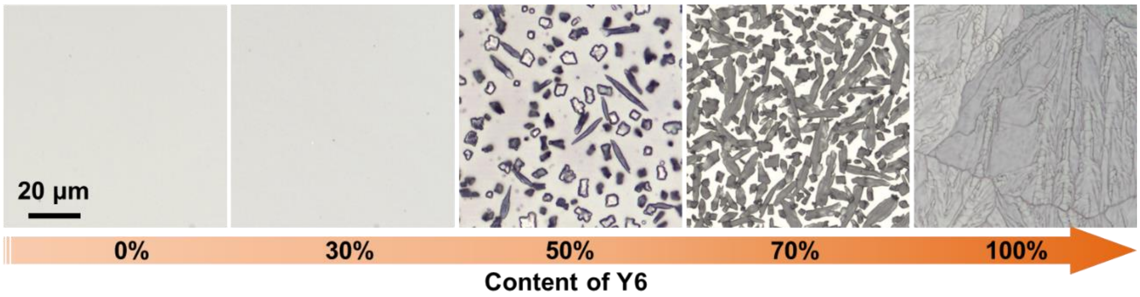

Supplementary Fig. 6 Optical micrographs of 30-min annealed PBDB-T:Y6 films in different proportions.

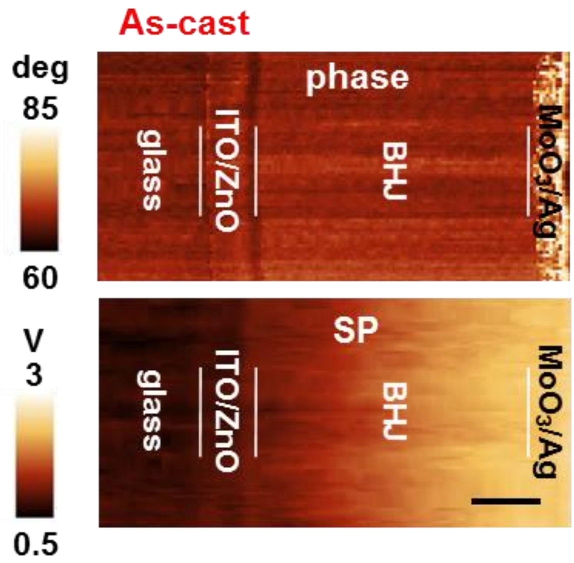

Supplementary Fig. 7 Phase and surface potential (SP) images of the as-cast device cross-section under illumination (AM 1.5G) at +2 V obtained by AFM (scale bar: 200 nm).

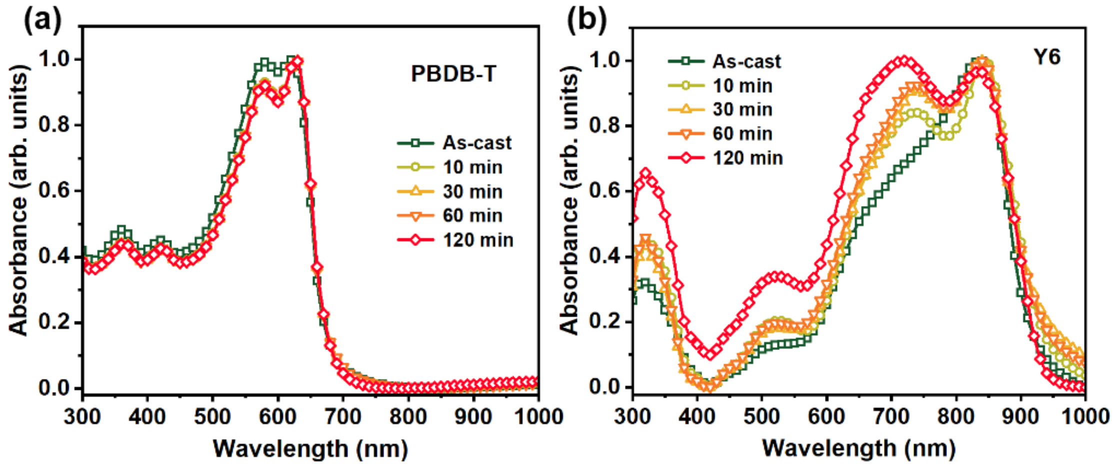

Supplementary Fig. 8 Normalized UV-vis-NIR absorption spectra of **a**, PBDB-T and **b**, Y6 films with different annealing durations.

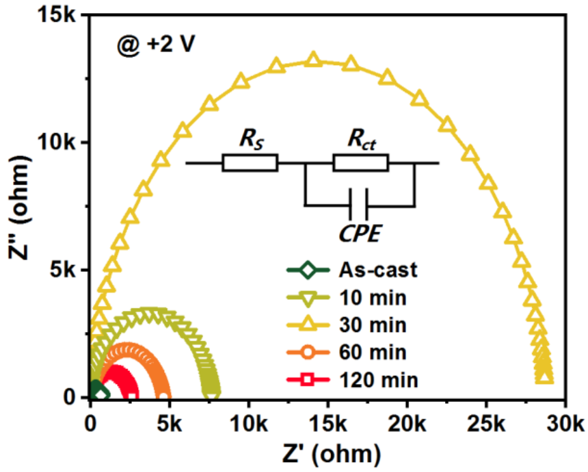

Supplementary Fig. 9 Nyquist plots of the 150-nm-thick OPDs with different annealing durations in dark condition (+2 V) and the equivalent electronic circuit (inset).

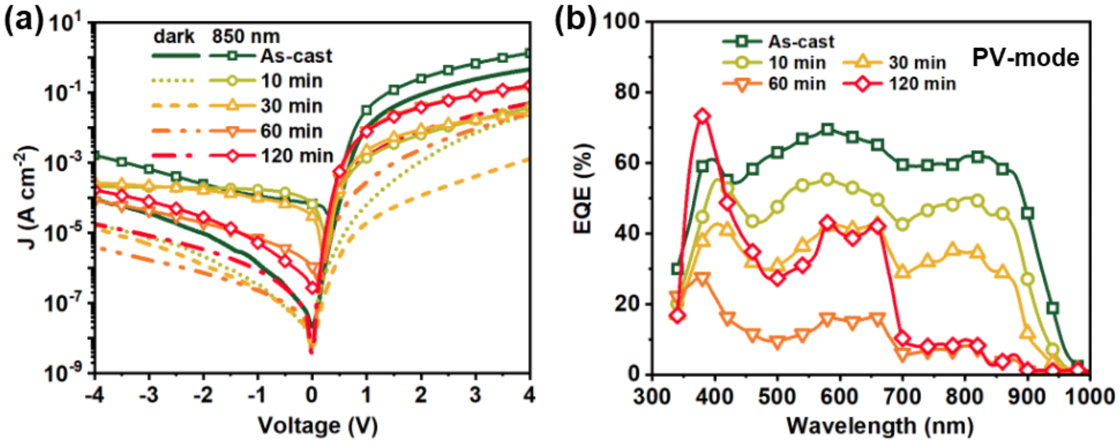

Supplementary Fig. 10 a *J-V* curves in dark and under 850 nm illumination of 0.737 mW cm^{-2}. **b** EQE spectra of 150-nm-thick OPDs with different annealing durations in PV-mode (-2 V).

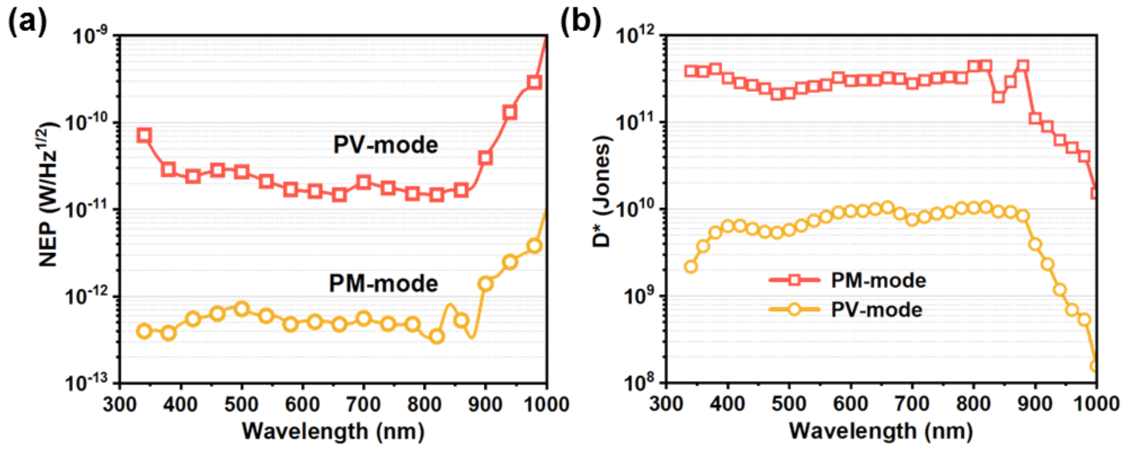

Supplementary Fig. 11 a NEP spectra, and **b** *D** spectra derived from actual noise (60 Hz) of 150-nm-thick 30-min-annealed OPD in PM and PV modes (±2 V).

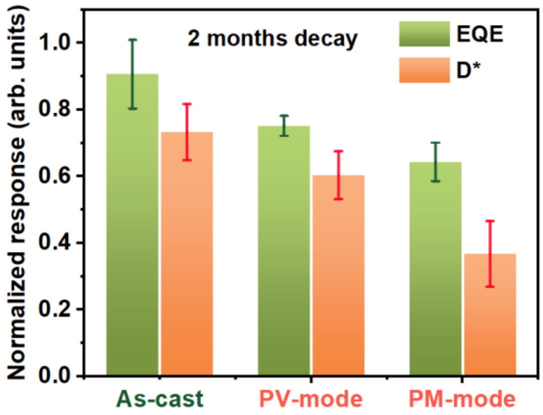

Supplementary Fig. 12 Durability of the as-casted and 30-min-annealed OPDs (N=5).

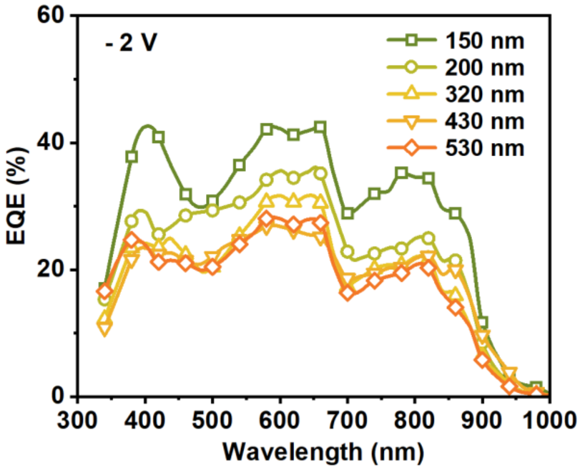

Supplementary Fig. 13 EQE spectra of 30-min-annealed OPDs with different BHJ thicknesses in PV-mode.

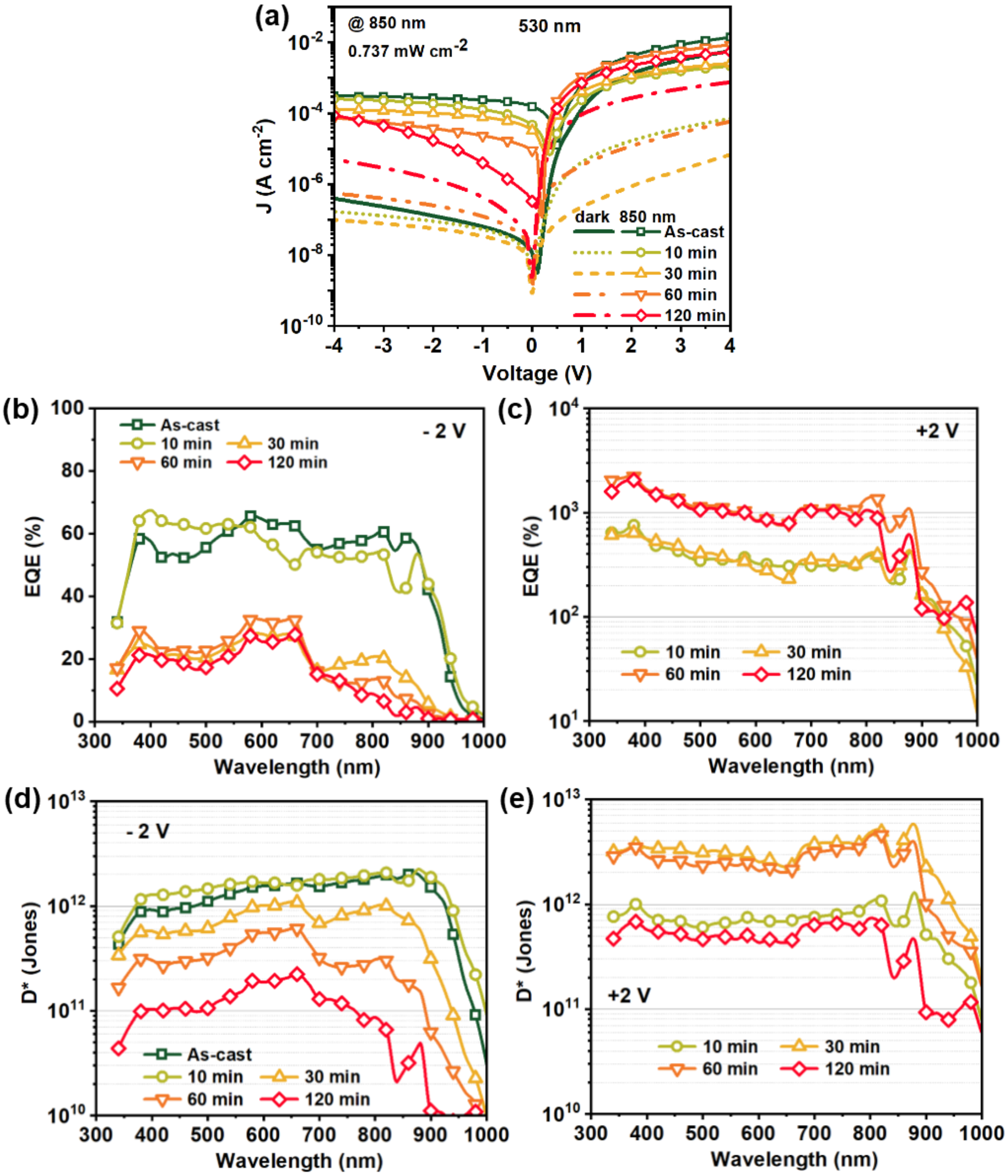

Supplementary Fig. 14 Performance of 530-nm-thick OPDs with different annealing durations: **a** *J*-*V* curves in dark and under 850 nm illumination of 0.737 mW cm^{-2}. **b-c** EQE spectra, and **d-e** D^* spectra in PM and PV modes (±2 V), respectively.

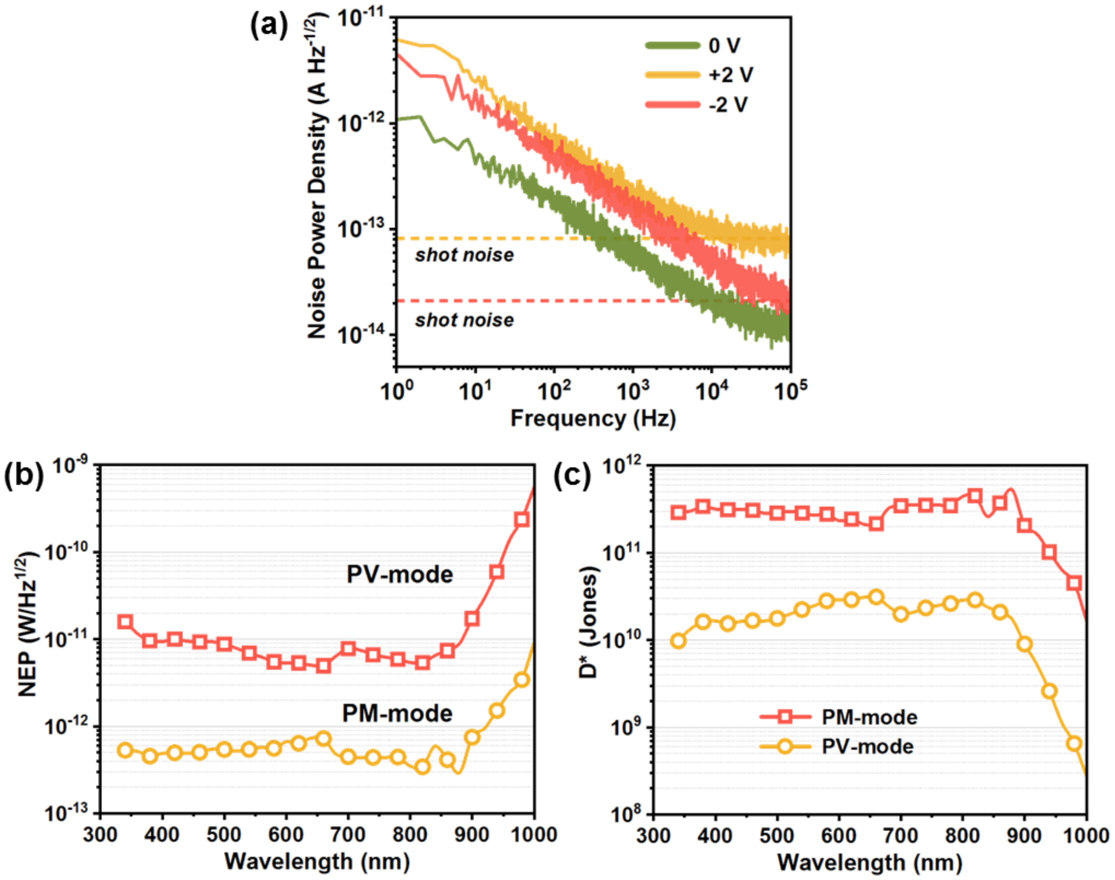

Supplementary Fig. 15 **a** Noise spectral density, **b** NEP and **c** S_n-derived (60 Hz) D^* spectra of 530-nm-thick OPDs in PM and PV modes (±2 V).

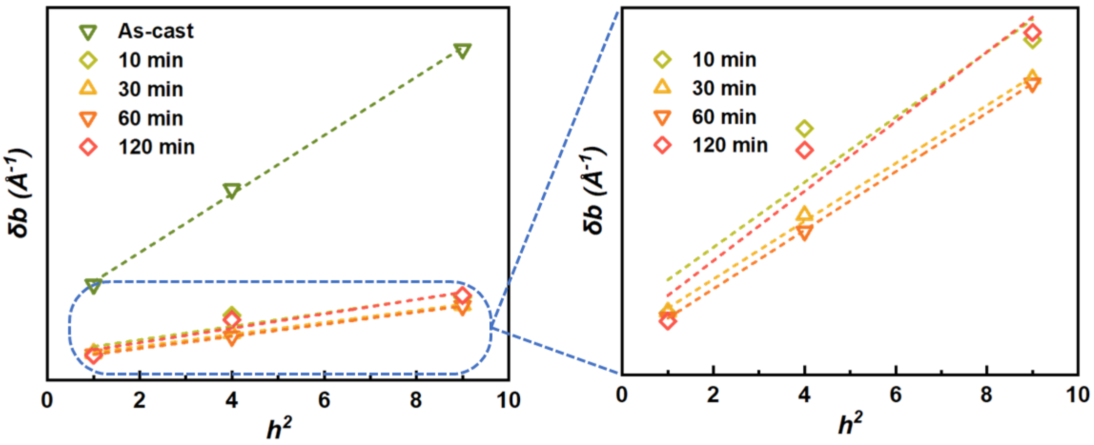

Supplementary Fig. 16 The δb - h^2 plot of the BHJ films extracted from in-plane line-cut profiles and the corresponding zoom-in plot.

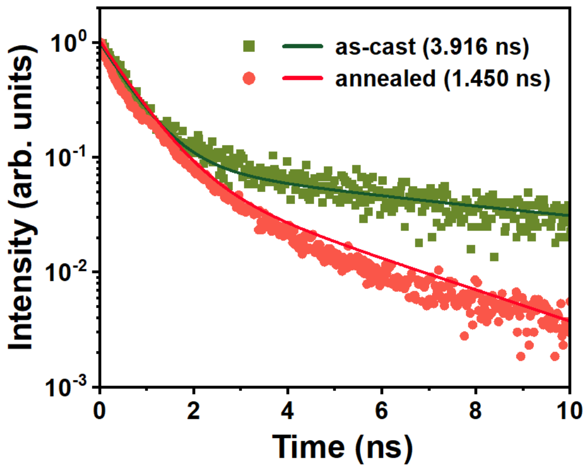

Supplementary Fig. 17 Time-resolved photoluminescence (TRPL) spectra of as-cast and 30-min-annealed BHJ films.

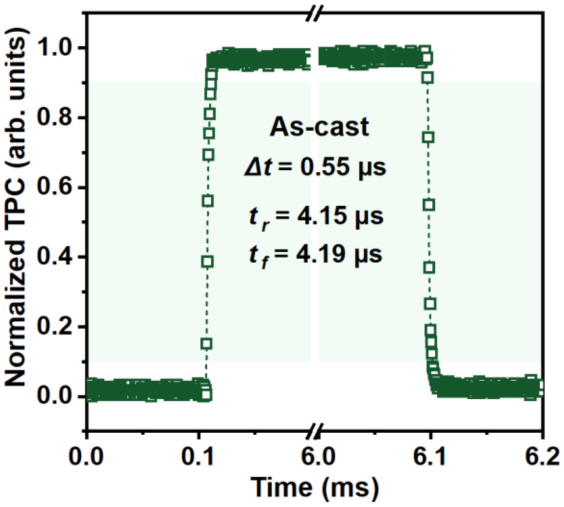

Supplementary Fig. 18. Response time of the 150 nm-thick as-cast OPD under incident 850 nm optical signal at -2 V.

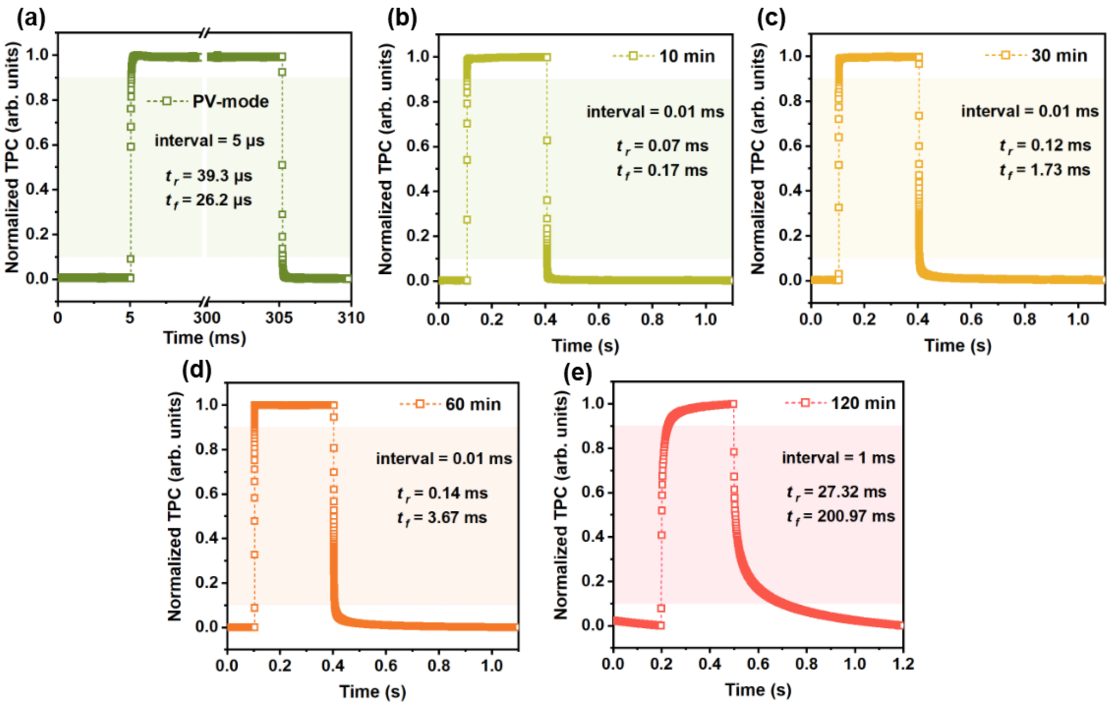

Supplementary Fig. 19 Response time of the 530 nm-thick devices under incident 850 nm optical signal. **a** TPC curve of the 30-min-annealed device in PV-mode at -2 V. **b-e** TPC curves of the devices with different annealing durations in PM-mode at +2 V.

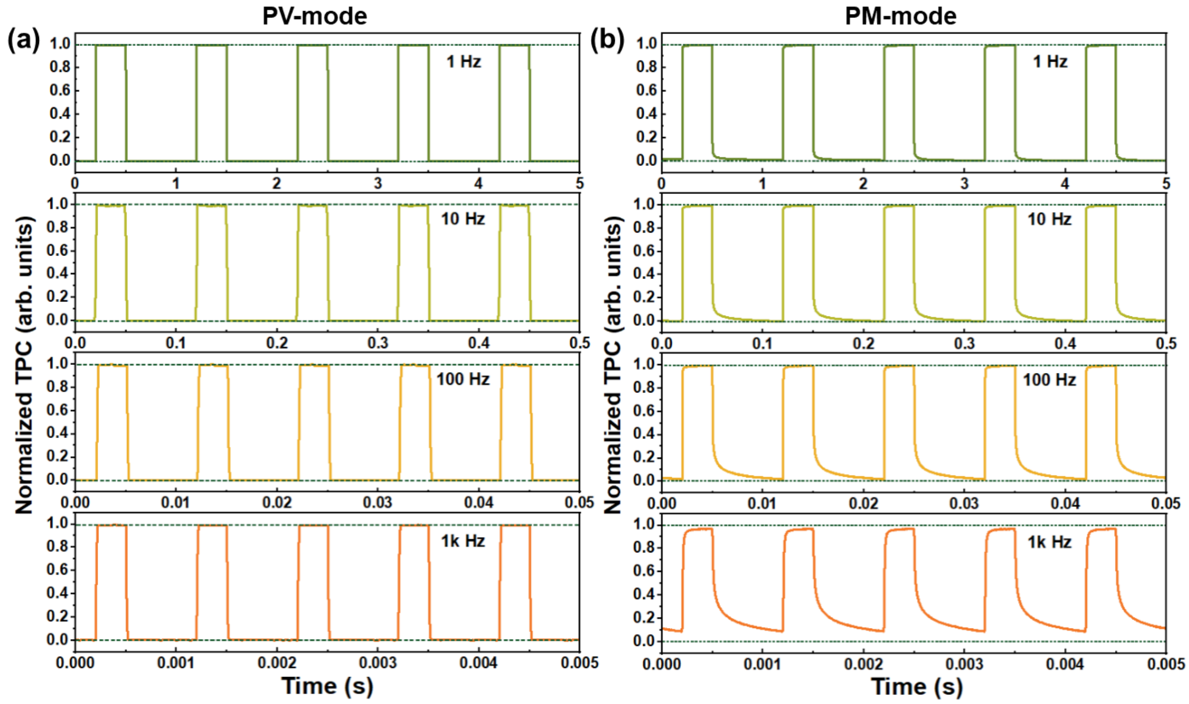

Supplementary Fig. 20 Response performance of the 150 nm-thick 30-min-annealed device under incident 850 nm continuous pulse signal in **a** PV-mode (-2 V) and **b** PM-mode (+2 V).

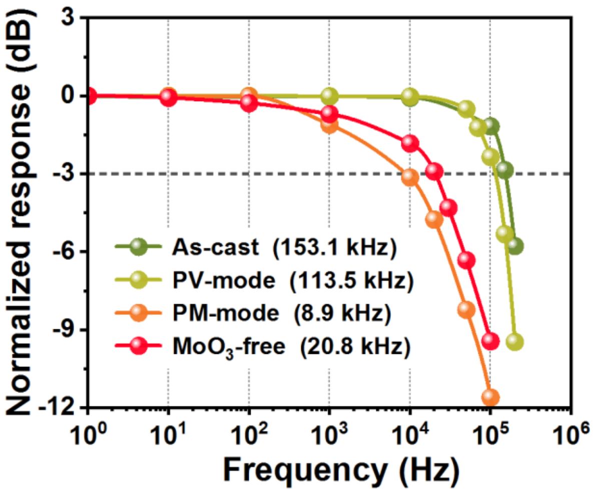

Supplementary Fig. 21 The -3dB cutoff frequency of the as-cast OPD (-2 V), dual-mode OPD operating in PV (-2 V) and PM (+2 V) modes, and 30-min-annealed MoO_3-free OPD (+2 V) under 850 nm light.

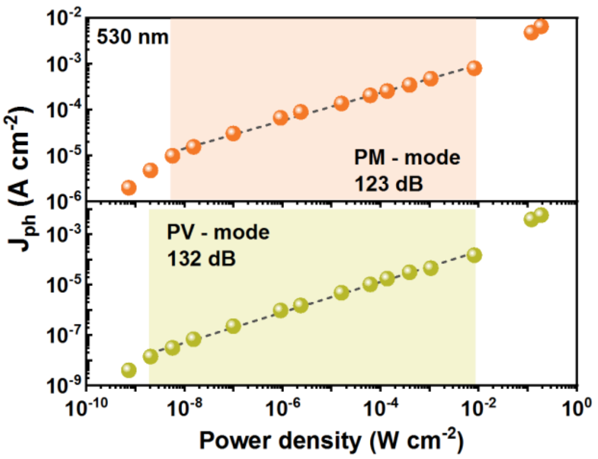

Supplementary Fig. 22 Power dependence of the 530-nm-thick 30-min-annealed device in PM and PV modes (±2 V).

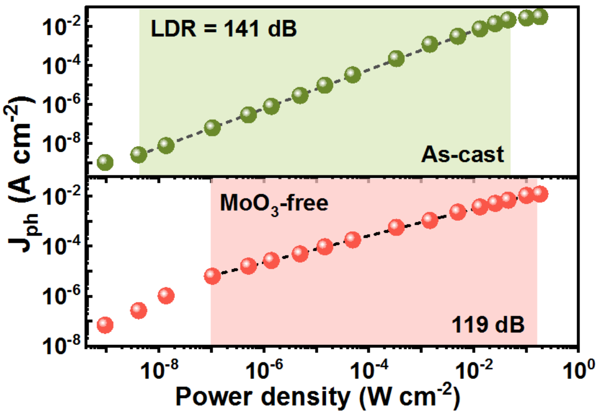

Supplementary Fig. 23 Power dependence of the 150 nm-thick as-cast OPD (-2 V) and 30-min-annealed MoO_3-free OPD (+2 V).

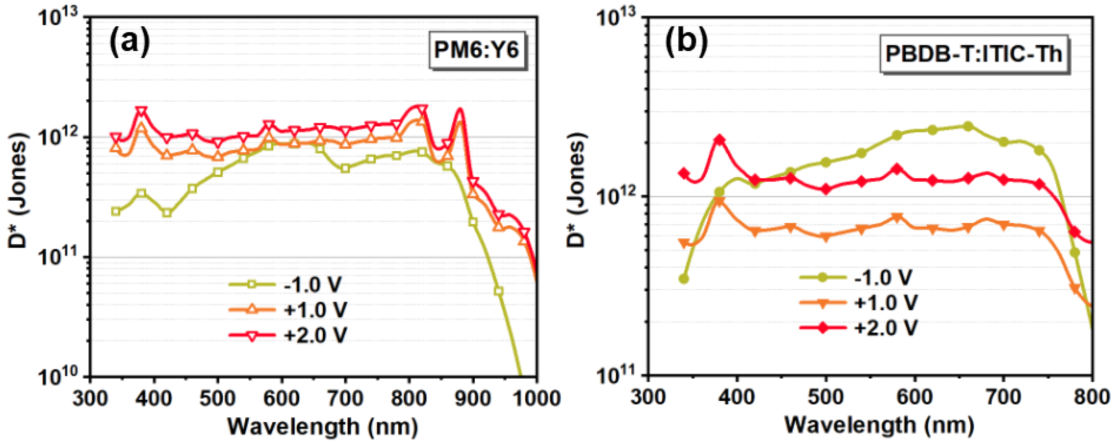

Supplementary Fig. 24 *D** spectra of the dual-mode (a) PM6:Y6 and (b) PBDB-T:ITIC-Th OPDs under different biases.

Supplementary Tables

Supplementary Table 1 Summary of GIWAXS parameters of BHJ films annealed for different durations.

	In-plane (lamellar stacking)			Out-of-plane (π-π stacking)		
	**d (nm)**	**Lc (nm)**	**$g_{(h00)}$ (%)**	**d (nm)**	**Lc (nm)**	**$g_{(010)}$ (%)**
As-cast	2.20	6.74	21.75	0.37	2.37	14.47
	1.01	3.97				
	0.69	2.48				
10 min	2.13	13.59	10.26	0.36	3.40	12.03
	0.98	8.70				
	0.71	7.41				
30 min	2.13	13.53	9.68	0.36	3.50	11.87
	0.98	10.46				
	0.71	7.92				
60 min	2.13	13.76	9.73	0.36	3.40	12.04
	0.98	10.87				
	0.71	7.99				
120 min	2.13	13.92	10.61	0.37	3.26	12.39
	0.98	9.08				
	0.71	7.32				

Supplementary Table 2 Performance comparison of recently reported PM OPDs.

Device Structure	Spectral range (nm)	D* (Jones) @ bias (V)	t_r/t_f (ms)	Ref
ITO/ZnO/PBDB-T:Y6/ MoO_3/Ag	340-1000	4.23×10^{12} @ 2 V	0.05/3.03	This work
ITO/PEIE/P3HT:$PC_{71}BM$ (100:1)/PEDOT:PSS/P3HT/NaF/Al	300-700	1.48×10^{14} @ -20 V	-/1140	1
ITO/TAPC/BT-ATPEPH:C_{60}/MoO_3:C_{70}/BCP/Ag	350-850	3.08×10^{12} @ -6 V	1.69/10	2
ITO/PEDOT:PSS/ P3HT:ETBI/Al	400-850	9.5×10^{12} @ −20 V	2.2/4.1	3
ITO/PEDOT:PSS/ PBDB-T:ITIC-Br/Al	400-1000	9.1×10^{11} @ -20 V	17/23	4
ITO/ZnO/D18-Cl:Y6-1O/ P3HT:$PC_{71}BM$/Au	350-750	3×10^{12} @ 5 V	0.72/0.91	5
ITO/PEDOT:PSS/poly-TPD:P3HT:$PC_{61}BM$/Al	300-900	9.1×10^{11} @ -20 V	51/20	6
ITO/PEDOT:PSS/PDBD-FBT:ITIC/Ag	300-800	3.82×10^{12} @ -10 V	0.5/3	7

Supplementary References

1. Wu, Y. L., Fukuda, K., Yokota, T. & Someya, T. A Highly Responsive Organic Image Sensor Based on a Two-Terminal Organic Photodetector with Photomultiplication. *Adv. Mater.* **31**, 1903687 (2019).
2. Guo, D.C. et al. Structure design and performance of photomultiplication-type organic photodetectors based on an aggregation-induced emission material. *Nanoscale* **12**, 2648-2656 (2020).
3. Yoon, S. et al. End-Group Functionalization of Non-Fullerene Acceptors for High External Quantum Efficiency over 150000% in Photomultiplication Type Organic Photodetectors. *Adv. Funct. Mater.* **31**, 2006448 (2020).
4. Wang, J. et al. Enhanced photomultiplication of organic photodetectors via phosphorescent material incorporation. *J. Mater. Chem. C* **9**, 16918-16924 (2021).
5. Kim, J., Kang, M., Lee, S., So, C. & Chung, D.S. Interfacial Electrostatic-Interaction-Enhanced Photomultiplication for Ultrahigh External Quantum Efficiency of Organic Photodiodes. *Adv. Mater.* **33**, 2104689 (2021).
6. Yang, K. X. et al. Smart Strategy: Transparent Hole-Transporting Polymer as a Regulator to Optimize Photomultiplication-type Polymer Photodetectors. *ACS Appl. Mater. Interfaces* **13**, 21565-21572 (2021).
7. Kim, J. et al. A regioregular donor-acceptor copolymer allowing a high gain-bandwidth product to be obtained in photomultiplication-type organic photodiodes. *Mater Horiz* **8**, 276-283 (2021).
